# The associations of AI assisted training on sport performance among student athletes based on a dual path chain mediation model with the moderating role of psychological adaptability

**DOI:** 10.3389/fpsyg.2025.1695704

**Published:** 2026-01-29

**Authors:** Huiying Zhang, Junjun Sun

**Affiliations:** 1 Department of Leisure Sports, Kangwon National University, Samcheok, Republic of Korea; 2 School of Foreign Languages, Shandong Vocational and Technical University of International Studies, Rizhao, China

**Keywords:** AI-assisted training, chain mediation, psychological adaptability, sport performance, student-athletes

## Abstract

**Purpose:**

This study examines the associations between AI-assisted training and sport performance among student-athletes using a dual-path chain mediation framework. Specifically, it tests two hypothesized chain mediation pathways and examines the moderating role of psychological adaptability.

**Methods:**

A cross-sectional questionnaire survey was conducted among 600 student-athletes from universities in three Chinese provinces using convenience sampling. Structural equation modeling and moderated regression analysis were employed to analyze the data.

**Results:**

AI-assisted training was significantly associated with higher sport performance, with evidence of a primary association (β = 0.124, 95% CI [0.091, 0.156], *p* = 0.0017) as well as weak additional associations transmitted through two chain mediation pathways—Path 1 (β = 0.003, 95% CI [0.002, 0.006], *p* = 0.0010) and Path 2 (β = 0.007, 95% CI [0.004, 0.011], *p* = 0.0012). The overall association was β = 0.135 (95% CI [0.101, 0.166], *p* = 0.0017). In addition, psychological adaptability was positively associated with the strength of this relationship, as reflected by a significant interaction term (β = 0.115, 95% CI [0.091, 0.138], *p* < 0.001).

**Conclusion:**

AI-assisted training was associated with higher sport performance both directly and indirectly through two conceptual pathways—cognitive-skill and behavioral-psychological processes. Moreover, the strength of these associations varied as a function of athletes' psychological adaptability, indicating a significant moderating role.

## Background

With the diffusion of artificial intelligence (AI) in sport training, AI-assisted training delivers efficient movement feedback and personalized prescriptions through wearables, virtual reality, and intelligent training programs, and has been gradually incorporated into routine practice across competitive sport, university physical education, and professional fitness (Huang et al., 2024; Bodemer, 2023; Wang and Wang, 2024). However, technology does not automatically translate into performance gains; its effects depend on AI feedback being translated into athletes' cognitive–skill improvements and affective–control processes (e.g., higher technical skill and tactical cognition, and optimized sport confidence, sport self-efficacy, and sport focus; Feltz et al., 2008), which in turn guide better decisions and motor execution during practice and competition.

Prior research has primarily focused on the associations between AI-related technologies and sport performance, with comparatively limited attention to the psychological and behavioral processes that may structure these associations across multi-step, sequential pathways, as well as to the boundary conditions under which such relationships vary. Specifically, empirical evidence remains scarce regarding whether AI-assisted training is linked to sport performance through a dual chain-mediation framework that integrates cognitive–skill processes (e.g., technical skill, tactical cognition) and affective–control processes (e.g., self-efficacy, focus/confidence). In addition, it remains unclear whether psychological adaptability—an important individual resource in contexts of technological change and training-related stress—is associated with variation in the strength of both the direct and mediated relationships between AI-assisted training and sport performance (Martin and Marsh, 2008). Accordingly, we ask:

RQ1: Does AI-assisted training improve sport performance?
RQ2: Does it affect performance via two chain-mediation routes representing cognitive–skill and affective–control processes?
RQ3: Does psychological adaptability positively moderate the relationship between AI-assisted training and sport performance?

### AI-assisted training and sport performance

In recent years, the application of AI in competitive training has expanded rapidly, showing substantial potential to increase training efficiency, improve monitoring accuracy, and optimize training processes. AI-assisted training delivers more precise, scientific, and personalized support for athletes' daily practice through smart wearables, virtual reality systems, and training management programs (Oliver and Guiller, 2025). Compared with traditional approaches, AI systems enable real-time data collection and analysis, provide continuous training recommendations, and support long-term performance tracking, thereby fostering a “human–machine collaborative” training ecology (Novatchkov and Baca, 2013; Hammes et al., 2022).

Within the development of sport performance, the scientific rigor of training methods and the timeliness of feedback have been widely recognized as important factors associated with performance-related outcomes (Hardy et al., 2010). AI-based tools are closely aligned with these aspects: motion recognition and physiological monitoring are increasingly used to support movement-quality monitoring and training-related risk assessment, while automated data feedback facilitates timely adjustments and strategic refinements during practice. Although existing studies have examined AI applications across school physical education, high-performance sport, and fitness contexts, systematic empirical evidence regarding how AI-assisted training is associated with sport performance—particularly through psychological and behavioral pathways—remains limited, especially among university student-athletes with substantial developmental potential. Accordingly, this study conceptualizes AI-assisted training as a contextual training-related factor and examines its associations with overall sport performance among university student-athletes, while further exploring multi-path psychological and behavioral pathways within a theoretically informed analytical framework.

#### Comparative summary (elite athletes vs. university student-athletes)

Relative to the highly structured, technology-rich ecology surrounding elite athletes (periodized planning, interdisciplinary support, stable motivational climates), university student-athletes face dual academic–athletic demands, greater heterogeneity in baseline skills, and uneven resource access. This contrast creates greater room for improvement and makes individual differences more decisive for “who benefits more” from AI. In this context, AI feedback is more likely to yield linked gains along cognitive–skill (technical skill, tactical cognition) and affective/control (sport confidence, self-efficacy, focus) pathways (Williams and Ford, 2013; Memmert and Roca, 2019; Feltz et al., 2008). Focusing on this sample is therefore practically necessary and theoretically advantageous for identifying sequential mechanisms and boundary conditions of AI's effects.

#### Critical appraisal and study positioning

Beyond application-oriented descriptions, prior research shows several limitations: (i) cross-sectional or short, lab-based protocols that obscure multi-stage, sequential mechanisms; (ii) small, homogeneous elite samples limiting generalizability to student-athletes; (iii) an emphasis on main effects with insufficient modeling of dual chain mediation; (iv) under-attention to key moderators (e.g., psychological adaptability) that determine who benefits more; and (v) heavy reliance on self-reports or coach ratings, with inadequate control for load, injury status, or algorithmic rules. Building on existing evidence and citations, this study constructs and tests a dual chain-mediation from AI to performance and incorporates psychological adaptability as a moderator to clarify both how AI translates into performance and for whom it is most effective (Hardy et al., 2010; Novatchkov and Baca, 2013; Hammes et al., 2022; Williams and Ford, 2013; Memmert and Roca, 2019; Feltz et al., 2008).

### Chain mediation effects of technical skill level, tactical cognition, and sport confidence

#### AI-assisted training and technical skill level

As an advanced technological intervention, AI-assisted training is reshaping traditional sports training models and has become a vital driving force in enhancing athletes' technical skill levels. Through the use of AI technologies—such as smart wearable devices, motion capture systems, and virtual simulation training platforms—athletes can receive real-time, multidimensional, and precise monitoring and feedback on their technical movements (Lin and Wang, 2024). This high-frequency, data-driven feedback mechanism effectively compensates for the limitations of traditional coaching based solely on experiential judgment, enabling more scientific and accurate optimization of technical skills.

AI systems, through deep learning and big data analytics, develop personalized skill enhancement plans for athletes by dynamically adjusting the intensity and content of technical training based on individual physiological characteristics and historical training data (Wang et al., 2022). For instance, by analyzing indicators such as movement trajectories, muscle force distribution, and balance control, AI can assist athletes in correcting technical deviations, thereby improving the standardization and fluidity of movements. This data-driven, personalized technical intervention not only increases the efficiency of skill acquisition but also reduces ineffective training and the risk of potential injuries.

Moreover, the integration of virtual reality (VR) and augmented reality (AR) technologies enables athletes to engage in repeated training of complex skills within highly simulated environments. These immersive simulations enhance adaptability and performance under pressure, laying a solid foundation for the flexible application of skills in real competitive settings (Novatchkov and Baca, 2013). With the continuous integration of AI-assisted training, athletes' technical skill levels are steadily improved, providing a crucial foundation for the development of more advanced tactical cognition.

#### Technical skill level and tactical cognition

Improvements in technical skill level not only enhance athletes' sport-specific performance but also provide the necessary physical and cognitive foundation for the development of tactical cognition. Previous studies have suggested that proficient technical abilities help free up cognitive resources, allowing athletes to focus more on the dynamic changes in the competitive environment and the strategic behaviors of opponents (Williams and Ford, 2013). This release of cognitive capacity enables athletes to grasp the overall structure and tactical framework of a game at a higher level, thereby improving their ability to perceive and anticipate complex situations (Memmert and Roca, 2019).

In addition, improvements in technical abilities enhance athletes' capacity for strategic decision-making and flexible adaptation during actual competitions. Athletes with proficient technical skills are better equipped to adjust their tactical execution in response to situational changes, thereby increasing their tactical adaptability and reaction speed. A higher level of technical reserve also provides athletes with greater agency during tactical confrontations, enabling them to cope with uncertainty and external pressure by leveraging their technical advantages, ultimately enhancing their control over the overall rhythm of the game (Staiano et al., 2025).

The positive interaction between technical and tactical elements is not only evident in competitive settings but is also continuously reinforced throughout the long-term training process. As technical skill levels improve, athletes are able to engage in more intensive and complex tactical training scenarios. Through repeated tactical drills and situational simulations, they further enrich their tactical cognition structure and strategic application skills. This upward spiral deepens athletes' understanding of the integration between technique and tactics, thereby improving their tactical execution and overall competitive performance in real match contexts.

#### Tactical cognition and sport confidence

Enhancement in tactical cognition serves as a critical psychological foundation for the development of a stable and confident mental state in athletes (Roca et al., 2011). Athletes with a high level of tactical cognition are able to accurately interpret the flow of the game and anticipate opponents' intentions, thereby increasing their sense of control over the outcome through the strategic application of tactics. This heightened sense of control directly reinforces athletes' sport self-efficacy, enabling them to exhibit greater psychological resilience and emotional stability when facing complex competitive environments (Hepler and Feltz, 2012).

The positive expectations and successful experiences derived from tactical cognition serve as a key pathway for building sport confidence. Through successful tactical decision-making, athletes receive continuous positive feedback, which reinforces their positive self-evaluation and sense of competence (Bandura, 1997). This sense of competence motivates athletes to maintain firm beliefs in their abilities and pursue higher goals, even under intense competitive pressure, thereby enhancing their challenge motivation and psychological resilience.

Athletes with well-developed tactical cognition are often able to formulate preemptive strategies during competition, reducing emotional fluctuations and anxiety triggered by unexpected situations. Such emotional stability further strengthens their confidence, enabling them to remain calm and make decisive judgments in critical moments, thus improving their performance and success rates under pressure. The positive cycle between tactical cognition and sport confidence not only optimizes athletes' emotional states but also enhances their performance in high-stakes environments.

#### Sport confidence and sport performance

Sport confidence has a direct impact on athletes' performance during competition and serves as a vital psychological resource for enhancing athletic outcomes (Hardy et al., 2018). Athletes with high levels of confidence tend to maintain stronger motivation and greater emotional stability throughout competitions. This positive psychological state facilitates sustained attentional focus, efficient execution of complex skills, and accurate tactical decision-making during critical moments.

Enhanced sport confidence enables athletes to exhibit greater adaptability and stress resilience when facing challenging situations. This psychological advantage not only reduces the likelihood of technical errors but also strengthens their ability to recover from failure and setbacks, allowing for a quicker return to optimal performance levels (Feltz et al., 2008).

Over the course of long-term athletic development, confidence reinforces itself through a positive feedback loop. Confident athletes, through repeated successful experiences and positive self-evaluations, develop a stable sense of self-efficacy and an optimistic attributional style. These positive psychological traits further enhance their potential for high-level performance in future competitions, providing a solid psychological foundation for their athletic careers.

### Chain mediation effects of sport self-efficacy, physical fitness, and sport focus

#### AI-assisted training and sport self-efficacy

AI-assisted training enhances athletes' perception of their own capabilities through high-precision data collection and intelligent analytical systems. With real-time training feedback, athletes can clearly understand their performance in areas such as technical execution and physical condition. The presentation of such objective data enables individuals to more directly perceive changes in their own abilities, thereby fostering the development of sport self-efficacy (Najami and Ghannam, 2025).

Throughout the ongoing training process, AI systems set achievable sub-goals in stages and dynamically adjust training intensity based on individual performance. As athletes continuously meet these incremental targets, they accumulate successful experiences through data-driven feedback. These experiences of goal attainment gradually foster a positive perception of their own abilities, leading to higher expectations and greater confidence in future training tasks (Pajares and Schunk, 2005).

The virtual reality and situational simulation systems provided by AI-assisted training replicate high-intensity confrontations and complex competition scenarios, enabling athletes to repeatedly practice in environments that closely resemble actual matches. When athletes are able to complete training tasks under such challenging conditions, their recognition of their own capabilities is reinforced through direct experiential success (Akbaş et al., 2019).

Intelligent algorithms not only offer personalized training recommendations but also provide phased feedback that allows athletes to observe their progress in ability development. Through this process, athletes gradually form expectations and confidence in their capacity to take on challenging tasks. This strengthened ability perception lays a solid psychological foundation for subsequent improvements in physical fitness and overall athletic performance.

#### Sport self-efficacy and physical fitness

Athletes' positive perception of their own abilities plays a critical motivational role in physical training. When athletes believe they are capable of handling high-intensity workouts, they are more willing to actively confront physical challenges and demonstrate stronger training motivation and persistence (Pajares and Schunk, 2005). This positive ability perception encourages individuals to set higher-level goals during the process of improving physical fitness. Through repeated completion of demanding training tasks, athletes continuously enhance their physical capacity (Feltz et al., 2008).

During sustained physical training, athletes' belief in their own capabilities contributes to maintaining a high level of engagement and focus. According to self-determination theory (Deci and Ryan, 2013), perceived competence stimulates intrinsic motivation, enabling individuals to demonstrate greater persistence and self-regulation when facing the challenges and fatigue of physical training. This activation of intrinsic motivation is reflected not only in the frequency and intensity of training, but also in athletes' proactive pursuit of more scientific and efficient physical conditioning methods to further strengthen their physical foundation.

Athletes' active engagement in physical training continuously leads to physiological adaptations, including improvements in core fitness indicators such as endurance, strength, and explosive power (Pal et al., 2023). As physical fitness improves, athletes' perception of their bodily capabilities is further reinforced. This positive feedback loop helps sustain high-intensity training over time, providing both the physical foundation and physiological support necessary for enhancing sport focus in subsequent stages.

#### Physical fitness and sport focus

Physical fitness directly influences athletes' ability to sustain attention and exert cognitive control during training and competition. Adequate physical reserves provide a stable energy supply to the brain, supporting high levels of cognitive functioning and attentional focus (Tomporowski et al., 2008). In high-intensity competitive environments, a well-maintained physical condition effectively mitigates the depletion of cognitive resources caused by fatigue, reduces attentional lapses, and enhances athletes' ability to perceive and respond to critical situations (Hockey, 1997).

Athletes with a strong physical fitness foundation exhibit greater physiological tolerance during prolonged high-intensity activities. This physiological advantage plays a crucial role in maintaining emotional stability and cognitive flexibility in complex environments (McMorris and Hale, 2012). Improved physical fitness not only enhances endurance and fatigue resistance but also helps athletes better regulate emotional fluctuations, preventing anxiety, attentional lapses, and slowed decision-making caused by physical exhaustion.

A well-conditioned physical state provides athletes with sufficient physiological resources to remain responsive under high-pressure competitive conditions. This allows them to rapidly detect critical cues and effectively activate relevant skills and tactical strategies. The positive interaction between physiological and cognitive systems enables athletes to sustain concentration and maintain a stable competitive state during key moments, thereby creating favorable conditions for optimal sport performance (Chang et al., 2012).

#### Sport focus and sport performance

Sport focus is a critical psychological factor that determines the quality of task execution in competitive settings. In complex and dynamic competition environments, athletes with a high level of attentional focus can effectively regulate their attention by directing cognitive resources toward key tasks, target movements, and tactical execution. This allows them to maintain technical stability and respond swiftly to tactical demands (Wilson et al., 2009). According to Nideffer's (1976) theory of attentional control, the efficient allocation and maintenance of attentional focus enhances information processing and reduces judgment errors and motor mistakes caused by environmental distractions.

During competition, athletes are exposed to a large volume of dynamic information inputs, including opponents' movements, environmental changes, and tactical adjustments. Strong attentional focus enables athletes to quickly filter relevant information and respond tactically within a limited time, thereby maintaining stable technical performance (Weinberg and Gould, 2023). Research has shown that athletes with higher attentional stability tend to exhibit greater psychological control and execution efficiency during critical stages of competition, supporting sustained high-level performance under pressure (Janelle, 2002).

In addition, focus is closely related to emotional regulation and psychological recovery. When athletes are able to maintain a focused state during competition, they typically demonstrate better mental endurance and self-control. This ability to sustain attention allows individuals to quickly adjust after errors or unexpected events, minimizing the disruptive impact of emotional fluctuations on technical execution, and enhancing the continuity and efficiency of their overall performance (Moran, 2016).

#### Moderating role of psychological adaptability

The introduction of artificial intelligence technologies has brought unprecedented changes to sports training, while simultaneously posing new challenges to athletes' capacity for adaptation. Psychological adaptability—the ability to adjust one's cognition and emotions in response to technological changes and training-related stress—has been recognized as a key moderating factor influencing the effectiveness of technological interventions (Biesecker et al., 2013).

AI-assisted training optimizes the training process through complex data feedback and personalized recommendations. However, its high level of technological sophistication and data intensity often lead to increased cognitive load and psychological stress (Sweller, 1988). Athletes with high psychological adaptability are more likely to embrace new technologies, quickly adjust their cognitive strategies and training approaches, and effectively integrate AI feedback, thereby enhancing training outcomes (Shin et al., 2020).

In contrast, individuals with low adaptability may resist technological changes or experience heightened anxiety, which can reduce their acceptance of AI tools and limit the effectiveness of AI-assisted training. This disparity suggests that psychological adaptability may moderate the relationship between technological intervention and sport performance by influencing athletes' attitudes toward AI and their ability to utilize feedback effectively.

Although the moderating role of psychological adaptability has been examined in general technology adaptation research (Pulakos et al., 2000), its specific mechanisms in AI-assisted training contexts remain underexplored. Investigating this issue not only helps explain individual differences in the effectiveness of technological interventions but also provides a novel perspective for improving the practical application of intelligent training systems.

#### Research model and hypotheses

**Figure 1:**
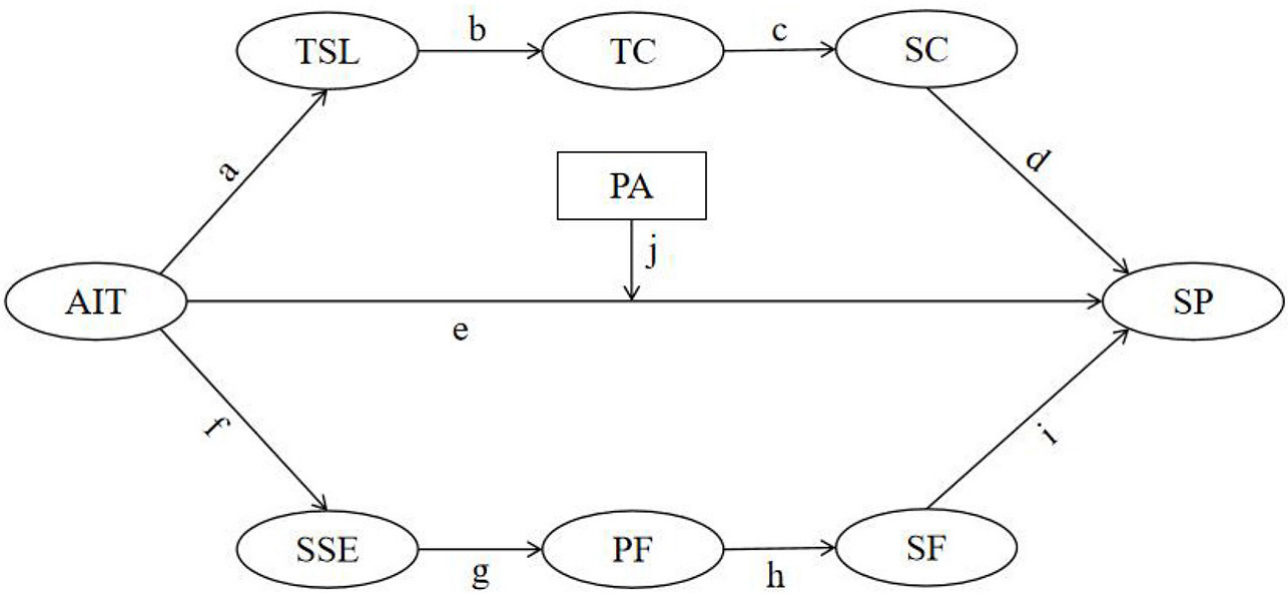
Conceptual model of chain mediation and moderation. AIT, AI-Assisted Training; TSL; Technical Skill Level; TC, Tactical Cognition; SC, Sport Confidence; SSE, Sport Self-Efficacy; PF, Physical Fitness; SF, Sport Focus; SP, Sport Performance; PA, Psychological Adaptability. H1 (effect “abcd”): AIT → TSL → TC → SC → SP; H2 (effect “e”): AIT → PS; H3 (effect “fghi”): AIT → SSE → PF → SF → SP; H4 (Moderation Path “j”): PA moderates the direct path AIT → SP.

Based on the preceding theoretical analysis and literature review, this study proposes a research model (Figure 1) to systematically examine the associations among AI-assisted training, sport performance, and related psychological and behavioral variables within a chain mediation and moderation framework. AI-assisted training is conceptualized as a training-related contextual factor, and its associations with sport performance are examined through two theoretically informed chain pathways: (1) a cognitive–skill pathway involving technical skill level, tactical cognition, and sport confidence; and (2) an action–control pathway consisting of sport self-efficacy, physical fitness, and sport focus. Furthermore, to account for individual differences in responses to technology-supported training contexts, psychological adaptability is incorporated as a moderating variable to examine whether the strength of the association between AI-assisted training and sport performance varies across levels of adaptability.

H1: AI-assisted training is positively associated with sport performance through a sequential association involving technical skill level, tactical cognition, and sport confidence, forming the chain pathway “AI-assisted training → technical skill level → tactical cognition → sport confidence → sport performance” (Path “a–b–c–d”).
H2: AI-assisted training is positively associated with sport performance after accounting for the sequential pathways (Path “e”).
H3: AI-assisted training is positively associated with sport performance through a sequential association involving sport self-efficacy, physical fitness, and sport focus, forming the chain pathway “AI-assisted training → sport self-efficacy → physical fitness → sport focus → sport performance” (Path “f–g–h–i”).
H4: Psychological adaptability moderates the association between AI-assisted training and sport performance, such that the association is stronger at higher levels of psychological adaptability (Path “j”).

## Methodology

### Sample size calculation

This study adopted a descriptive cross-sectional research design. The sample size was estimated using the Leslie Kish formula, which is appropriate for single proportion estimation (Kish, 1965):


$$\begin{array}{l} n=\frac{Z^{2}\cdot p\cdot \left(1-p\right)}{d^{2}}=\frac{1.96^{2}\times 0.5\times \left(1-0.5\right)}{0.04^{2}}\approx 600 \end{array}$$

*Z* is the critical value from the standard normal distribution at a 95% confidence level, taken as 1.96. The proportion *p* is set conservatively at 0.5 in the absence of prior information, because *p* (1–*p*) reaches its maximum value (0.25) when *p* = 0.5. This ensures that the sample size calculation is based on the “maximum variance” scenario, providing a more robust estimate in the field of sports psychology, where the proportion is unknown and the population is relatively heterogeneous, thus reducing the risk of Type II errors. The margin of error *d* is set at 0.04, meaning the estimation precision is controlled within ±4 percentage points. This choice falls within the commonly used range of 0.03–0.05 in practical research, balancing precision and feasibility: on one hand, it yields a sufficiently narrow confidence interval to capture subtle differences in behavioral or psychological measures, and on the other hand, it takes into account the practical constraints of sample recruitment and resource availability.

### Participants

This study employed a convenience sampling method to distribute the survey questionnaires. Traditional random sampling methods may result in low response rates and inaccurate answers. Although theoretically all studies should use random sampling, this is often nearly impossible in practice, especially for populations that are difficult to access. Convenience sampling offers advantages such as rapid implementation, low cost, and high flexibility. Therefore, a convenience sampling approach was adopted to recruit 636 student-athletes from universities in Liaoning, Heilongjiang, and Guangdong provinces in China. To ensure the quality of the questionnaire data, the study employed anonymous responses and paid participants 5 RMB each for completing the survey. A total of 636 questionnaires were distributed and collected through both paper-based surveys and the Wenjuanxing online platform. After excluding 36 invalid responses, including duplicate entries and answers deemed unreliable, 600 valid questionnaires were obtained, yielding a valid response rate of 94.34%. The deadline for data collection was March 15, 2025. We note that convenience sampling may introduce some limitations in sample representativeness, and the 5 RMB incentive—intended primarily as time compensation—may have introduced minor motivational differences. To mitigate these risks, we used anonymous responses and conducted quality screening. The impact on the main conclusions appears limited; future studies, where feasible, may adopt stratified random sampling for further validation.

All participants were Chinese student-athletes currently enrolled in university and aged 18 years or older. The average age was 20.8 years (SD = 1.6), reflecting typical characteristics of the university student population. Among the 600 student-athletes, 41.8% participated in basketball, 30.6% in badminton, 17.3% in table tennis, 6.6% in volleyball, and 3.5% in other sports. There were 295 male participants, accounting for 49.2%. The AI-assisted training tools used in this study included smartwatches and fitness bands (such as Apple Watch and HUAWEI Watch), virtual reality devices (head-mounted displays or gloves), fitness applications or programs (such as Keep, Codoon, and MyFitnessPal), and ChatGPT.

This study involved minimal-risk, anonymous, and non-invasive survey procedures. According to the ethical review policy of Shandong Foreign Languages Vocational College, the study was granted exemption from ethics approval. All procedures were conducted in accordance with the ethical standards of the Declaration of Helsinki. Informed consent was obtained from all participants prior to their participation.

### Measures

The measurement instruments used in this study were adapted from previously validated and well-established scales in prior research and were further modified in accordance with the theoretical framework and local contextual considerations.

AI-assisted training scale (3 items) was adapted from the AI-Assisted Attitude Scale for Chinese College English Learners developed by Wang et al. (2024). The psychological adaptability scale (4 items) was based on the Psychological Adaptation to Health Conditions Scale by Biesecker et al. (2013). The technical skill level scale was developed by adapting relevant constructs from Lin and Wang (2024), Jud and Thalmann (2025), and Oliver (2023). Similarly, the tactical cognition scale was constructed using theoretical insights from Wang et al. (2024), He et al. (2024), and Hsu et al. (2019). The sport confidence scale (3 items) was adapted from the Athlete Self-Efficacy Scale developed by Koçak (2020), while the sport self-efficacy scale (4 items) was adapted from the Health Self-Efficacy Scale by Schwarzer and Renner (2009). The physical fitness scale was developed based on conceptual frameworks from Wachholz et al. (2025), Singh et al. (2023), Skopal et al. (2024), and Phu et al. (2019). The sport focus scale (3 items) was adapted from the Athlete Mindfulness Questionnaire by Zhang et al. (2017), and the sport performance scale (7 items) was derived from the Test of Performance Strategies (TOPS) developed by Hardy et al. (2010).

The translation and localization of the scales followed internationally recognized procedures for cross-cultural adaptation, including forward translation, back-translation, expert panel review, and pilot testing. All items were rated on a 7-point Likert scale (1 = “strongly disagree,” 7 = “strongly agree”). Following Nunnally and Bernstein (1994) reliability standards, reliability analyses were conducted in SPSS; Cronbach's alpha coefficients ranged from 0.921 to 0.978, indicating excellent internal consistency (Table 2). Preliminary results suggest that the instruments exhibit high applicability and sound psychometric properties for university student-athlete populations.

### Data analysis

Multiple statistical methods were employed in this study to ensure the reliability and validity of the measurement model and to systematically examine the significance of the dual chain mediation and the moderating effect of psychological adaptability.

First, to control for potential common method bias (CMB), Harman's single-factor test was conducted. All measurement items were entered into an unrotated factor analysis using SPSS. If the variance explained by the first factor is less than 40%, it indicates that no serious common method bias exists.

Second, to assess the construct validity of the measurement instruments, confirmatory factor analysis (CFA) was conducted using AMOS 26.0. Structural models were built between latent variables and observed variables, and standardized factor loadings, composite reliability (CR), and average variance extracted (AVE) were calculated to evaluate convergent and discriminant validity within the theoretical model.

Third, after confirming the structural validity of the scales, descriptive statistics were performed using IBM SPSS 26.0, including means, standard deviations, maximum and minimum values. Skewness and kurtosis were tested to assess normality, and all variables were found to meet the assumptions of normal distribution, providing a basis for subsequent model testing.

Next, Pearson correlation analysis was conducted to examine the relationships among AI-assisted training, technical skill level, tactical cognition, sport confidence, sport self-efficacy, physical fitness, sport focus, and sport performance. This analysis served to preliminarily assess linear relationships and check for multicollinearity issues among variables.

To further test the statistical significance of the dual chain mediation pathways, structural equation modeling (SEM) was conducted using AMOS 26.0. The bias-corrected non-parametric bootstrap method with 5,000 resamples was employed to test the indirect effects of the mediation model, thereby establishing the dual mediation mechanisms and clarifying the transmission pathways leading to sport performance.

Finally, to examine the moderating role of psychological adaptability, the PROCESS macro (Model 1) in SPSS was used to conduct multiple regression analyses. The significance of the interaction term was tested to assess whether the association between sport self-efficacy and sport performance varied as a function of psychological adaptability. The significance level was set at *p* < 0.05. Given the cross-sectional self-report design, the effects estimated from the structural equation model are interpreted as model-consistent statistical associations rather than evidence of causal processes.

## Results

### Common method bias and model fit testing

To examine whether common method bias was present in the data, two complementary approaches were employed.

First, Harman's single-factor test was conducted by entering all measurement items from the study variables simultaneously into an unrotated exploratory factor analysis. The results showed that the first factor accounted for only 18.456% of the total variance, which is significantly below the critical threshold of 40% (Podsakoff et al., 2003).

Second, a single-factor confirmatory factor analysis was performed using AMOS, following the procedure recommended by Podsakoff et al. (2003). In this analysis, all measurement items were constrained to load onto a single latent factor. The results showed a poor model fit (χ^2^ = 16,905.390, *df* = 902, χ^2^*/df* = 18.742, GFI = 0.299, AGFI = 0.231, CFI = 0.307, NFI = 0.296, TLI = 0.273, RMSEA = 0.172), failing to meet the model fit criteria proposed by Hu and Bentler (1999) (χ^2^*/df* ≤ 3, GFI, AGFI, CFI, NFI, and TLI ≥ 0.90, RMSEA ≤ 0.08). This indicates that a single-factor model cannot adequately account for the covariance among the measurement items.

Subsequently, a full measurement model including all latent variables was estimated. As shown in Table 1, the model demonstrated an excellent fit to the data (χ^2^ = 982.542, *df* = 893, χ^2^*/df* = 1.100, GFI = 0.932, AGFI = 0.924, CFI = 0.996, NFI = 0.959, TLI = 0.996, RMSEA = 0.013).

**Table 1:** Model fit indices.

***χ^2^***	***df***	***χ^2^/df***	**GFI**	**AGFI**	**CFI**	**NFI**	**TLI**	**RMSEA**
982.542	893	1.1 < 3	0.932 > 0.9	0.924 > 0.9	0.996 > 0.9	0.959 > 0.9	0.996 > 0.9	0.013 < 0.06

χ^2^/df: ratio of chi-square to degrees of freedom; smaller is better; < 3 typically indicates good fit (< 5 acceptable). GFI, Goodness-of-Fit Index, an absolute fit measure; ≥ 0.90 good (≥0.95 better). AGFI, Adjusted GFI, accounts for model complexity; ≥0.90 good. CFI, Comparative Fit Index, an incremental fit measure; ≥0.90 good (≥0.95 better). NFI, Normed Fit Index, an incremental fit measure; ≥0.90 good. TLI, Tucker–Lewis Index/NNFI, incremental fit with a penalty for complexity; ≥0.90 good (≥0.95 better). RMSEA, Root Mean Square Error of Approximation; smaller is better; ≤ 0.06 good (0.06–0.08 acceptable).

Taken together, the results from Harman's single-factor test and the AMOS single-factor analysis provide consistent evidence that common method bias does not pose a serious threat to the validity of the findings in this study.

### Confirmatory factor analysis

Based on the first-order measurement model, CFA was conducted for all measured variables. Following the recommendation of Fornell and Larcker (1981), measurement items with factor loadings below 0.60 were removed, and the final measurement model was constructed. The CFA results are presented in Table 2.

**Table 2:** Confirmatory factor analysis (CFA) results.

**Latent variable**	**Observed variable**	***Unstd*.**	***S.E*.**	***Z***	***p***	***Std*.**	**SMC**	**CR**	**AVE**	**Cronbach's α**
AIT	AIT1	1				0.900	0.810	0.929	0.814	0.921
	AIT2	1.003	0.031	32.521	^***^	0.902	0.814			
	AIT3	1.308	0.040	32.693	^***^	0.905	0.819			
PA	PA1	1				0.916	0.839	0.939	0.794	0.938
	PA2	0.999	0.030	33.439	^***^	0.882	0.778			
	PA3	1.015	0.034	30.073	^***^	0.843	0.711			
	PA4	1.018	0.027	37.283	^***^	0.922	0.850			
TSL	TSL1	1				0.787	0.619	0.929	0.567	0.929
	TSL2	0.986	0.047	20.782	^***^	0.779	0.607			
	TSL3	0.948	0.047	20.296	^***^	0.765	0.585			
	TSL4	0.934	0.048	19.666	^***^	0.745	0.555			
	TSL5	0.916	0.047	19.655	^***^	0.745	0.555			
	TSL6	0.929	0.048	19.410	^***^	0.738	0.545			
	TSL7	0.953	0.048	19.816	^***^	0.750	0.563			
	TSL8	0.922	0.047	19.626	^***^	0.744	0.554			
	TSL9	0.958	0.047	20.340	^***^	0.766	0.587			
	TSL10	0.871	0.047	18.419	^***^	0.707	0.500			
TC	TC1	1				0.743	0.552	0.921	0.565	0.921
	TC2	1.024	0.055	18.538	^***^	0.754	0.569			
	TC3	1.015	0.055	18.333	^***^	0.746	0.557			
	TC4	1.03	0.055	18.605	^***^	0.756	0.572			
	TC5	1.056	0.056	18.811	^***^	0.764	0.584			
	TC6	1.068	0.055	19.450	^***^	0.788	0.621			
	TC7	1.025	0.055	18.639	^***^	0.758	0.575			
	TC8	1.000	0.055	18.034	^***^	0.735	0.540			
	TC9	0.956	0.054	17.567	^***^	0.717	0.514			
SC	SC1	1				0.920	0.846	0.925	0.805	0.925
	SC2	0.974	0.031	31.531	^***^	0.876	0.767			
	SC3	1.013	0.031	32.750	^***^	0.895	0.801			
SSE	SSE1	1				0.895	0.801	0.942	0.803	0.942
	SSE2	1.022	0.03	34.337	^***^	0.911	0.830			
	SSE3	1.039	0.03	34.922	^***^	0.918	0.843			
	SSE4	1.011	0.034	30.077	^***^	0.859	0.738			
PF	PF1	1				0.871	0.759	0.954	0.806	0.954
	PF2	1.030	0.033	30.822	^***^	0.887	0.787			
	PF3	1.036	0.032	32.188	^***^	0.905	0.819			
	PF4	1.027	0.031	33.484	^***^	0.922	0.850			
	PF5	1.046	0.033	31.953	^***^	0.902	0.814			
SF	SF1	1				0.941	0.885	0.947	0.857	0.947
	SF2	0.992	0.024	41.149	^***^	0.927	0.859			
	SF3	0.973	0.025	39.038	^***^	0.909	0.826			
SP	SP1	1				0.929	0.863	0.978	0.864	0.0.978
	SP2	0.998	0.021	46.839	^***^	0.951	0.904			
	SP3	1.001	0.023	43.170	^***^	0.931	0.867			
	SP4	0.995	0.023	42.846	^***^	0.929	0.863			
	SP5	0.992	0.024	41.319	^***^	0.920	0.846			
	SP6	1.002	0.025	40.638	^***^	0.915	0.837			
	SP7	1.014	0.024	42.965	^***^	0.930	0.865			

^***^p < 0.001.

The analysis showed that the standardized factor loadings ranged from 0.707 to 0.951 and were all statistically significant, indicating strong associations between the observed indicators and their corresponding latent constructs. The CR values ranged from 0.921 to 0.978, exceeding the commonly accepted threshold of 0.70 and suggesting excellent internal consistency and measurement stability across constructs. It is worth noting that some constructs had CR values above 0.95, which may theoretically indicate potential item redundancy; in response, we re-examined item semantics and loading patterns and found no obvious duplication, although future studies may consider streamlining items—while preserving content validity—to further enhance measurement efficiency. In addition, the AVE ranged from 0.565 to 0.864, indicating that most of the variance in the observed variables was well explained by their respective latent variables, thereby demonstrating good convergent validity.

In summary, the measurement model used in this study demonstrated excellent structural reliability and validity, providing a solid foundation for subsequent structural equation modeling analyses.

### Correlation and descriptive statistical analysis

The mean values (M) of all latent variables ranged from 3.97 to 4.44, indicating that participants generally held positive perceptions toward each construct. The standard deviations (SD) ranged from 0.80 to 1.70, suggesting a moderate level of variability in the responses. The absolute values of skewness ranged from 0.02 to 0.09, and those of kurtosis ranged from 0.58 to 0.98, all within the recommended thresholds (skewness < 2, kurtosis < 8), indicating that the data approximately followed a normal distribution Kline, (2023).

Additionally, to assess discriminant validity, this study employed the AVE-based method for correlation and descriptive statistical analysis. As shown in Table 3, the square root of the AVE for each latent variable (bolded along the diagonal) was greater than the inter-construct correlation coefficients, indicating adequate discriminant validity among the variables Miao and Zhang, (2024). Taken together, these findings provide strong empirical support for the structural integrity, reliability, and validity of the proposed measurement model.

**Table 3:** Correlations, descriptive statistics, and square roots of AVEs.

**Latent variable**	**AIT**	**PA**	**TSL**	**TC**	**SC**	**SSE**	**PF**	**SF**	**PS**
AIT	***0.902***								
PA	0.106^***^	***0.891***							
TSL	0.370^***^	0.066^*^	***0.753***						
TC	0.140^***^	−0.001	0.264^***^	***0.752***					
SC	0.038^*^	0.004^*^	0.078^*^	0.332^***^	***0.897***				
SSE	0.363^***^	0.095^**^	0.115^***^	0.046^*^	0.044^*^	***0.896***			
PF	0.121^***^	−0.003	−0.043	0.002^*^	0.005^*^	0.304^***^	***0.898***		
SF	0.077^*^	0.006^*^	0.085^**^	0.027^*^	−0.026^*^	0.073^*^	0.277^***^	***0.926***	
PS	0.339^***^	0.012^*^	0.176^***^	0.100^**^	0.191^***^	0.118^***^	0.138^***^	0.504^***^	***0.930***
*M*	4.44	4.00	4.00	3.97	3.99	4.00	3.98	4.01	4.03
*SD*	1.70	1.48	1.15	1.14	1.15	1.18	1.12	1.10	0.80
*Skew*	−0.02	−0.09	0.04	0.04	0.03	−0.06	−0.07	0.02	0.05
*Kurtosis*	−0.93	−0.98	−0.64	−0.73	−0.78	−0.58	−0.66	−0.82	0.59
*AVE*	0.81	0.79	0.57	0.57	0.81	0.80	0.81	0.86	0.86

^***^p < 0.01, ^**^p < 0.01, ^*^p < 0.05. Italicized bold values on the diagonal represent the square roots of AVEs.

Correlation analyses indicated that AI-assisted training (AIT) was significantly and positively associated with the key mediators and the outcome variable, including technical skill level (TSL) (*r* = 0.370, *p* < 0.001), sport self-efficacy (SSE) (*r* = 0.363, *p* < 0.001), and sport performance (PS) (*r* = 0.339, *p* < 0.001). The critical associations along the hypothesized chain pathways were also significant, such as TSL–tactical cognition (TC) (*r* = 0.264, *p* < 0.001), TC–sport confidence (SC) (*r* = 0.332, *p* < 0.001), SSE–physical fitness (PF) (*r* = 0.304, *p* < 0.001), and PF–sport focus (SF) (*r* = 0.277, *p* < 0.001). Notably, SF exhibited the strongest correlation with PS (*r* = 0.504, *p* < 0.001), providing preliminary support for the subsequent structural model tests.

### Chain mediation effect analysis

**Figure 2:**
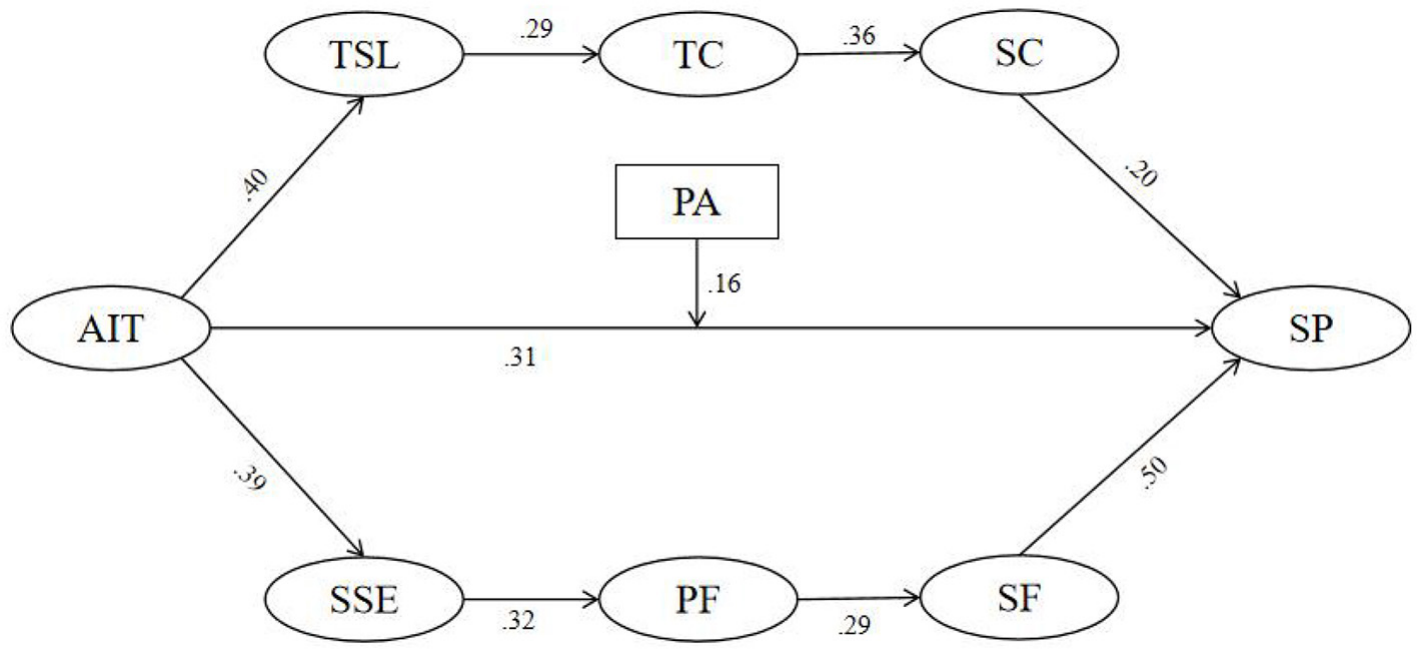
Chain mediation path model with standardized path coefficients.

Figure 2 presents the standardized path coefficients of the chain mediation model. To ensure comprehensive reporting, the unstandardized path coefficients derived from the AMOS output are also provided. The path from AI-assisted training to technical skill level (Path “*a”*) was significant (γ = 0.243, *p* < 0.001), as was the path from technical skill level to tactical cognition (Path “*b”*, γ = 0.265, *p* < 0.001), from tactical cognition to sport confidence (Path “*c”*, γ = 0.371, *p* < 0.001), and from sport confidence to sport performance (Path “*d”*, γ = 0.143, *p* < 0.001). Additionally, the direct path from AI-assisted training to sport performance (Path “*e”*) remained significant (γ = 0.124, *p* < 0.001). Regarding the second chain mediation pathway, the path from AI-assisted training to sport self-efficacy (Path “*f”*) was significant (γ = 0.226, *p* < 0.001), as were the subsequent paths from sport self-efficacy to physical fitness (Path “*g”*, γ = 0.312, *p* < 0.001), from physical fitness to sport focus (Path “*h”*, γ = 0.275, *p* < 0.001), and from sport focus to sport performance (Path “*i”*, γ = 0.377, *p* < 0.001). These results provide empirical support for the hypothesized dual chain mediation mechanisms linking AI-assisted training to sport performance.

This study employed SEM to analyze and test the chain mediation effects, thereby improving estimation accuracy. The bootstrap method was used to estimate the standard errors of the indirect effects and assess their statistical significance. As shown in Table 4, the total effect of the model was 0.135, with a standard error (SE) of 0.016. The Z-value, calculated as the ratio of the effect to its standard error (*Z* = 0.135/0.016), was 8.438, exceeding the critical threshold of 1.96. In addition, the 95% confidence interval (CI) [0.101, 0.166] did not include zero, indicating that the total effect was statistically significant (*p* < 0.01).

**Table 4:** Chain mediation effect analysis based on bootstrap estimates.

**Hypothesis**	**Model**	**Estimate**	**Product of coefficients**		***95% CI***		***p***
			***S.E***.	***Z***	**Lower**	**Upper**	
H1	Path “abcd”	0.003	0.001	3.000	0.002	0.006	0.0010^**^
H2	Path “e”	0.124	0.017	7.294	0.091	0.156	0.0017^**^
H3	Path “fghi”	0.007	0.002	3.500	0.004	0.011	0.0012^**^
	Total	0.135	0.016	8.438	0.101	0.166	0.0017^**^

^**^p < 0.01.

Specifically, the chain mediation effect along Path H1 (effect “abcd”) was estimated at 0.003, with a standard error of 0.001, yielding a Z-value of 3.000. The 95% CI [0.002, 0.006] excluded zero, and the result was statistically significant (*p* = 0.0010), indicating that the chain mediation effect along H1 was significant.

Practical implications (based on unstandardized coefficients): Holding other variables constant, each 1-unit increase in AI-assisted training (AIT) is associated with an average increase of 0.135 units in sport performance (SP) (95% CI [0.101, 0.166]). Of this, the direct effect is about 0.124; the combined indirect effect through the two chain-mediation paths is about 0.010 (H1 = 0.003; H3 = 0.007), accounting for approximately 7.4% of the total effect. In standardized terms, a 1 SD increase in AIT corresponds to an increase of about 0.31 SD in SP, indicating a medium effect size. These findings suggest that strengthening the intensity and quality of AIT in practice can not only directly enhance sport performance but also yield additional gains via the two mechanisms of “technical skill level → tactical cognition → sport confidence” and “sport self-efficacy → physical fitness → sport focus.” For target back-calculation, under the assumptions of scale range and local linearity, if the desired improvement in SP is Δ units, AIT would need to increase by approximately Δ/0.135 units, providing a reference for coaches and administrators when setting intervention intensity.

Although both sequential indirect effects were statistically significant, their magnitudes were small (H1 = 0.003; H3 = 0.007). The combined indirect effect (0.010) accounted for approximately 7.4% of the total effect (0.135), indicating that the AIT–SP association was driven primarily by the direct effect, with the proposed chains providing modest incremental explanatory value.

### Moderation effect analysis

To examine the moderating effect of psychological adaptability on the relationship between AI-assisted training and sport performance, this study constructed a regression model including an interaction term (i.e., X × W → Y) without incorporating mediator variables. Specifically, psychological adaptability and its interaction with AI-assisted training were simultaneously entered into the regression analysis to test the moderating effect of psychological adaptability on the association between AI-assisted training and sport performance. To further elucidate the form of this moderation effect, simple slope analyses were conducted based on the significant interaction effect, and the corresponding interaction plots were generated and interpreted.

According to Table 5, the overall model demonstrated a good fit [*F*_(3, 596)_ = 60.511, *p* < 0.001], explaining 23.4% of the total variance in sport performance (*R*^2^ = 0.234). AI-assisted training had a significant positive predictive effect on sport performance (β = 0.166, *t* = 9.764, *p* < 0.001, 95% CI [0.133, 0.199]). The moderator variable, psychological adaptability, did not exhibit a significant direct effect on sport performance (β = −0.001, *t* = −0.044, *p* > 0.05, 95% CI [−0.039, 0.038]). Importantly, the interaction term between AI-assisted training and psychological adaptability (H4) significantly predicted sport performance (β = 0.115, *t* = 9.586, *p* < 0.001, 95% CI [0.091, 0.138]), indicating that psychological adaptability plays a significant positive moderating role in this relationship, thus supporting Hypothesis 4.

**Table 5:** Moderation effect analysis.

**Variable**		**Dependent variable: sport performance**			
		β	***SE***	***t***	***95% CI***
Independent variable	AI-assisted training (*a*)	0.166^***^	0.017	9.764	[0.133, 0.199]
Moderator variable	Psychological adaptability (*b*)	−0.001	0.020	−0.044	[−0.039, 0.038]
Interaction effect	Interaction (*a × b*, Path *j*)	0.115^***^	0.012	9.586	[0.091, 0.138]
	*R*	0.483			
	*R^2^*	0.234			
		F_(3, 596)_ = 60.511^***^			

^***^p < 0.001. The interaction effect supports H4.

**Figure 3:**
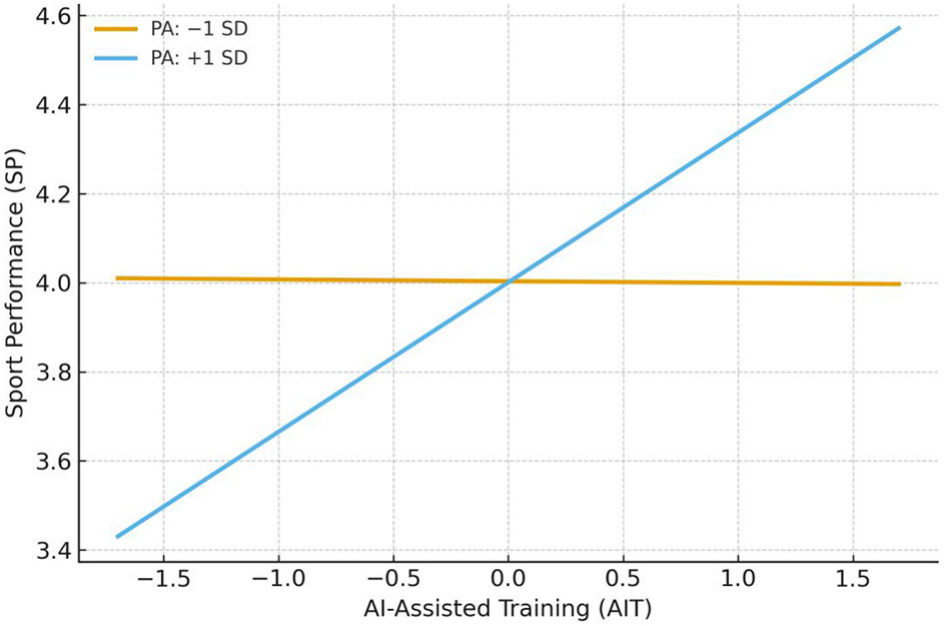
Simple slopes of AIT predicting SP at levels of PA.

Simple slopes for the moderating effect (Table 6; Figure 3). When psychological adaptability was low (−1 SD), the conditional effect of AIT on SP was not significant (β = −0.004, SE = 0.024, *t* = −0.159, 95% CI [−0.051, 0.044]). This indicates that among individuals with low psychological adaptability, AI-assisted training is unlikely to translate into a clear performance gain; although the point estimate was negative, the confidence interval crossed zero, preventing any inference of a true negative effect.

**Table 6:** Comparison of moderating effects at high and low levels of psychological adaptability.

**Psychological adaptability**	**β**	***SE***	***t***	***95% CI***
Low (−1 SD)	−0.004	0.024	−0.159	[−0.051, 0.044]
High (+1 SD)	0.336^***^	0.025	13.463	[0.287, 0.385]

^***^p < 0.001.

By contrast, when psychological adaptability was high (+1 SD), the conditional effect of AIT on SP was positive and significant (β = 0.336, SE = 0.025, *t* = 13.463, 95% CI [0.287, 0.385], *p* < 0.001). That is, under higher psychological adaptability, the positive association between AI-assisted training and sport performance is markedly strengthened, supporting the hypothesis that psychological adaptability positively moderates the direct AIT → SP path.

## Discussion

The findings suggest that AI-assisted training is positively associated with sport performance among university student-athletes, with the association being driven primarily by the direct link to performance and only modestly reflected in two parallel sequential pathways. Specifically, these small indirect associations were observed through a cognitive–skill pathway and an action–psychological pathway, indicating that AI-related training experiences are linked not only to observable training processes but also to athletes' cognitive appraisals and the mobilization of psychological resources.

In addition, the moderating role of psychological adaptability highlights meaningful individual differences in how strongly AI-assisted training is associated with sport performance. The results indicate that this association varies with athletes' capacity to engage with and internalize feedback from AI-based training systems. This pattern is consistent with perspectives on technological adaptability and points to practical implications for the personalization and optimization of AI-supported training environments.

**H1. Technical skill** **→** **tactical cognition** **→** **sport confidence (chain mediation)**

AI-assisted training significantly predicted sport performance through a sequential mediation pathway involving enhanced technical skill, improved tactical cognition, and elevated sport confidence. The significance of this chain mediation effect validates the dynamic linkage among cognitive, skill-based, and emotional dimensions, thereby supporting Hypothesis H1.

This finding not only confirms the direct effectiveness of AI in improving technical capabilities but, more importantly, reveals its indirect empowerment of athletes' psychological mechanisms. It suggests that AI training does not merely influence the quality of overt physical execution, but may also generate a cascade of psychological effects involving cognitive processing, strategic judgment, and emotional mobilization. Particularly in systematic training contexts, this stepwise mechanism facilitates the formation of a positive feedback loop—“skill enhancement → cognitive clarity → increased confidence → improved performance.” This underscores the structural and developmental potential of AI-assisted systems in optimizing sport performance outcomes.

The findings of this study resonate with the framework proposed by Huang et al. (2024), who described the three-phase functionality of AI in sports coaching as “guidance–monitoring–correction.” According to their study, AI technologies enhance training precision and efficiency by providing real-time data analysis and personalized feedback, thereby improving the systematic development of athletic skills and offering a solid foundation for cognitive processing. Similarly, He et al. (2024) emphasized in their study on AI-based tactical analysis models that tactical cognition depends not only on experiential learning but also heavily on data modeling and situational simulation enabled by AI. By mapping scenarios and providing visualized decision support, AI systems enable athletes to grasp complex tactical structures more swiftly, thereby enhancing both cognitive complexity and strategic flexibility. The psychological implications of tactical cognition have also received increasing attention. Iftode and Ursu (2024) found that athletes employing positive cognitive-emotional regulation strategies exhibited stronger sport confidence and psychological resilience during competition.

It is worth noting, however, that the current findings diverge somewhat from Jud and Thalmann (2025) systematic review, which concluded that AI has limited effects on emotional variables among athletes. Their review, based primarily on elite-level professional athletes, posited that such individuals possess more stable psychological structures that are less susceptible to change via technological intervention. In contrast, the present study focuses on student-athletes, whose psychological development remains relatively malleable. As such, they may be more receptive to feedback and cognitive guidance, which facilitates the internalization of confidence and perceived control during skill acquisition.

**H2. Direct effect: AI-assisted training** **→** **sport performance**

AI-assisted training was found to exert a direct effect on sport performance, independent of any mediating variables. This finding highlights the technological pathway through which AI directly enhances athletic performance by optimizing skill execution, improving training efficiency, and regulating physiological states. By leveraging smart devices and data-driven systems, AI-based training enables real-time feedback on technical movements and physical conditions, allowing athletes to make immediate adjustments and improve performance outcomes (Oliver, 2023).

This direct effect is consistent with the findings of Tang et al. (2025), who developed a real-time monitoring and analysis system for track and field athletes based on edge computing and deep reinforcement learning algorithms. Their system provided continuous feedback on athletes' movement quality and physical status, enabling rapid and targeted adjustments that improved both the consistency and quality of athletic performance.

Similarly, Najami and Ghannam (2025) demonstrated the effectiveness of VR technologies in tennis training. By integrating wearable sensors with immersive VR environments, athletes received immediate feedback on variables such as racket swing speed and force, which significantly improved movement consistency and strategic decision-making. These findings further support the direct facilitative role of AI technologies in enhancing athletic skill performance.

**H3. Sport self-efficacy** **→** **physical fitness** **→** **sport focus (chain mediation)**

AI-assisted training positively predicted sport performance through a sequential mediation pathway involving enhanced sport self-efficacy, improved physical fitness, and elevated sport focus. This finding supports the validity of a multi-level chain pathway of “cognitive appraisal → physical capacity → attentional regulation.”

AI training systems, through personalized feedback and progressive reinforcement, help athletes develop a more positive perception of their abilities, thereby significantly enhancing sport self-efficacy. This result aligns with Ma et al. (2025) assertion that self-efficacy is positively associated with training motivation. Richlan et al. (2023) also noted that individuals with high self-efficacy are more likely to set challenging goals and maintain consistent physical training efforts, which ultimately lead to better physical condition.

Within this mediation pathway, physical fitness acts as a pivotal node connecting cognitive and attentional mechanisms. Pompa et al. (2024) argued that improved physical fitness reduces the cognitive depletion caused by physiological fatigue, thereby supporting sustained attention. Wachholz et al. (2025) further emphasized that enhanced physical condition promotes emotional regulation and cognitive flexibility, enabling athletes to maintain attentional stability even under high-intensity training conditions.

Sport focus serves as a critical psychological regulator within this pathway. Attention control directly influences the quality of skill execution and tactical performance. As Weinberg and Gould (2023) highlighted, effectively managing attentional focus can reduce distractions and enhance information processing efficiency. AI-assisted training, by providing real-time feedback and high-fidelity simulation environments, facilitates greater attentional engagement and task immersion, ultimately contributing to improved sport performance.

**H4. Psychological adaptability as a moderator of AIT** **→** **SP**

In the relationship between AI-assisted training and sport performance, psychological adaptability emerged as a significant moderating variable. This finding indicates that individuals differ in their capacity to receive and internalize external feedback when exposed to technological interventions, and such individual differences may influence the effectiveness of AI-based training. According to Wachholz et al. (2025), psychological adaptability is a crucial psychological resource that enables individuals to cope with environmental changes and stressful conditions, closely associated with their ability to integrate external information. Individuals with higher psychological adaptability are more likely to revise cognitive appraisals and positively adopt feedback and recommendations provided by AI systems.

This observation aligns with the Unified Theory of Acceptance and Use of Technology (UTAUT) proposed by Venkatesh et al. (2003), which highlights that users' perceptions of the usefulness and ease of use of a technology significantly influence their willingness to adopt it. Psychologically adaptable individuals typically possess greater cognitive flexibility and emotional regulation capacity, making them more inclined to embrace technological change. Judge and Bono (2001) emphasized that core self-evaluation traits—such as self-esteem, self-efficacy, locus of control, and emotional stability—are closely related to job satisfaction and performance. These traits may also shape an individual's openness to technological innovations. In the context of AI-assisted training, this means that individuals with strong psychological adaptability are better able to convert external feedback into internal behavioral adjustments, thus refining their training strategies and skill development trajectories.

Furthermore, based on the coping strategy theory of Carver and Connor-Smith (2010), individuals with higher psychological adaptability tend to exhibit more effective emotional and cognitive coping mechanisms when encountering pressure or changes in the environment. This adaptive capacity not only affects how individuals interpret and respond to feedback but also contributes to subsequent performance regulation and psychological recovery during training. In AI-assisted training, psychological adaptability moderates the cognitive processing of feedback and behavioral responses, indirectly influencing training outcomes and changes in sport performance.

Based on this study, first classify psychological adaptability into low, medium, and high using within-team norms, then tailor AI-assisted training accordingly. For low psychological adaptability, build the base by lowering task difficulty, raising success rates, and using delayed or summative feedback on a few key points to strengthen self-efficacy and stability. For medium psychological adaptability, progress steadily by adding contextual tasks and technical variations while maintaining technical quality, and combine real-time feedback with key-frame replays to sharpen tactical judgment. For high psychological adaptability, emphasize integration and competition by increasing task complexity and time and space pressure, use multi-metric real-time feedback, and schedule a deload week every 3 to 4 weeks for consolidation and recovery. Monitor weekly completion rate, technical stability, situational accuracy, and attentional focus; if athletes with low psychological adaptability show no improvement over 2 weeks, prioritize psychological or attention training rather than simply increasing volume. For goal setting, use the estimated slope as a rough guide: to raise sport performance by X units, increase AI-assisted training by approximately X divided by 0.135 units, provided the measurement scale is appropriate, local linearity holds, and the athlete's load tolerance is acceptable.

## Limitations and future directions

Several limitations should be noted. First, the cross-sectional design limits causal inference; longitudinal or intervention studies are needed to test sustained effects. Second, the regionally concentrated sample (northeastern and southern China) may constrain generalizability; future work should recruit larger, more diverse samples and examine heterogeneity via multi-group analyses (e.g., sport type, gender, personality traits, and populations). Third, sport performance was self-reported, potentially introducing common-method and social desirability biases; objective indicators (skill tests, match statistics, coach ratings, and wearable-derived metrics) should be incorporated. Finally, while current AI systems excel in real-time feedback and movement correction, personalized psychological support remains limited; integrating emotion recognition, cognitive regulation, and individualized interventions may enhance adaptive value.

## Conclusion and recommendations

This study systematically explored the impact pathways of AI-assisted training on the sport performance of university student-athletes using chain mediation and moderation models. The results demonstrated that AI-assisted training not only indirectly influenced sport performance by enhancing technical skill, tactical cognition, and sport confidence, but also exerted effects through a dual cognitive–action pathway involving sport self-efficacy, physical fitness, and attentional focus. Furthermore, psychological adaptability played a significant moderating role in the relationship between AI training and sport performance, reflecting individual differences in the effectiveness of technological interventions. Therefore, the core conclusion of this study can be summarized as “two chain mediations and one moderator”: AI-assisted training enhances sport performance through two pathways—technical skill → tactical cognition → sport confidence and sport self-efficacy → physical fitness → attentional focus—and the strength of this effect is significantly moderated by psychological adaptability.

Based on these findings, it is recommended that university sports training programs actively adopt AIs-powered devices and personalized training systems to improve training efficiency and athletic performance through real-time technical feedback. At the same time, efforts should be made to cultivate athletes' psychological adaptability, enhancing their acceptance of and responsiveness to emerging technologies, thereby maximizing the benefits of AI-assisted training. Future AI training systems should strengthen capabilities in emotion recognition and personalized feedback, and align training approaches with athletes' psychological characteristics and developmental stages. This will enable more adaptive and targeted smart training solutions, facilitating the integration of technology and personalization in modern sports training.

## Data Availability

The datasets presented in this study can be found in online repositories. The names of the repository/repositories and accession number(s) can be found in the article/supplementary material.
